# 

**Published:** 1879-05

**Authors:** 


### CONTENTS:

ARTICLE I.—The Conservative Treatment of the Dental Pulp. By Richard Grady, D. D. S. .... 1
ARTICLE II.—From a Preliminary Lecture by Prof. Hodgkin. .... 20
ARTICLE III.—Micrology.—No. 1. By Geo. B. Harriman, D. D S., M. D. .... 25
ARTICLE IV.—Micrology.—No. 2. By Geo. B. Harriman, D. D. S., M. D. .... 27
ARTICLE V.—Evils Arising from Daily Use of Astringent Mouth Washes. By J. N. Farrar, M. D. .... 30
ARTICLE VI.—Animal Grafting—Its Therapeutical Application to Certain Lesions of the Dental Apparatus. By Dr. E. Magitot .... 34
ARTICLE VII.—Privilege Tax of Dentists. By S. P. Cutler, M. D., D. D. S .... 37
ARTICLE VIII.—Dental Associations—National Dental Association .... 38
New England Dental Societies .... 40
Georgia State Dental Society .... 40

### EDITORIAL, ETC.

Death of Josiah Bacon .... 40
Remarks on Article on Foul Breath .... 45
Dr. Adolph Petermann .... 46
A Batch of Dentists .... 47
Annual Meeting of Alumni of Baltimore College of Dental Surgery .... 48
A New Department .... 48

### BIBLIOGRAPHICAL.

Dr. Petermann's Dental Almanac for 1879 .... 48

